# Generative AI tool use enhances academic achievement in sustainable education through shared metacognition and cognitive offloading among preservice teachers

**DOI:** 10.1038/s41598-025-01676-x

**Published:** 2025-05-13

**Authors:** Javed Iqbal, Zarqa Farooq Hashmi, Muhammad Zaheer Asghar, Muhammad Naseem Abid

**Affiliations:** 1 School of English Studies, Zhejiang International Studies University, Hangzhou, People’s Republic of China; 2 School of Education, Huazhong University of Science and Technology, Wuhan, People’s Republic of China; 3 Department of Education, University of Helsinki, Helsinki, 0014 Finland; 4 Learning Educational and Technologies (LET) Research Lab, University of Oulu, Oulu, Finland

**Keywords:** Generative artificial intelligence tool usage, Academic achievement, Shared metacognition, Cognitive offloading, Preservice teachers, Psychology, Environmental social sciences

## Abstract

The integration of generative artificial intelligence tools in education has emerged as a transformative approach to enhancing learning outcomes, particularly in the context of sustainable development goals (SDG4). Therefore, the present study investigates the connection between generative artificial intelligence tool usage (GenAITU) and academic achievement (AA) in the context of SDG4. We assessed the mediating role of shared metacognition (SMC) and cognitive offloading (COL) in this relationship among preservice teachers (PSTs). The indicators, including performance expectancy (PE), effort expectancy (EE), facilitating conditions (FC), and use behavior (UB), are derived from adapting the Unified Theory of Acceptance and Use of Technology 2 (UTAUT2) for GenAITU. The authors surveyed 465 students from five universities in Wuhan, China, using a 7-point Likert scale through a time-lag design. Statistical analysis was performed through partial least squares structural equation modeling (PLS-SEM), to determine the relationship between variables. Findings indicated that two components of GenAITU, namely PE and UB, showed significant positive associations with AA, while the other two, EE and FC, did not show significant and positive relationships with AA. Results also showed that three dimensions of GenAITU, namely EE, FC, and UB have a positive and significant association with SMC while PE has a positive and significant connection with SMC. All four components of GenAITU like PE, EE, FC, and UB have positive and significant links with COL. SMC and COL have a positive and significant relationship with AA. Results also indicated that SMC mediated the connections between GenAITU (EE, FC, and UB) and AA. Outcomes also indicated that COL mediated the connections between GenAITU (PE, EE, FC, and UB) and AA. The current study shows that SMC and COL were strong mediators of the association between GenAITU and AA. The results of our study provide guidance to teachers, curriculum planners, and university management to successfully integrate GenAITU into the education for PSTs.

## Introduction

Technological advancements and transformations have become powerful tools for opening new pathways to improve performance at both individual and organizational levels^1^. Higher education institutions are also evolving with these advancements to cope with the challenges of the 21st century by providing updated knowledge and skills to students^2^. GenAITU is pivotal in shaping the AA of future educators, particularly in alignment with SDG4, which focuses on inclusive and equitable quality education^3,4^. GenAITU has become crucial. Therefore, in this study, we introduced two critical factors, namely SMC and COL, that can be significantly influential when PST uses generative AI tools to enhance AA to achieve SDG4 ^5–8^.

In recent years, the integration of GenAITU into educational environments has rapidly transformed the ways in which learners interact with digital technologies^9^. GenAITU behavior refers to the manner in which individuals, particularly pre-service teachers (PSTs), engage with, adopt, and utilize generative AI applications to support their academic and pedagogical tasks. Within the framework of UTAUT2, constructs such as PE, EE, FC, and UB serve as critical indicators of this behavioral engagement^10^. These tools’ use behavior offers novel opportunities for cognitive support, instructional planning, and content generation; however, their meaningful adoption in pedagogical contexts is still emerging^11–13^. Despite growing interest, limited empirical research has explored how PSTs perceive and interact with GenAITU and how such behavior relates to their AA. Therefore, our study explores how GenAITU behavior is essential to improve AA in the context of SDGs.

With this, AA represents a core outcome variable in educational research, reflecting learners’ performance, progress, and mastery of academic content^14^. In the context of PSTs, AA not only indicates individual success but also serves as a predictor of future teaching effectiveness and professional readiness. As digital tools like GenAITU become more prevalent, understanding their impact on AA is increasingly important^15^. Emerging constructs such as SMC and COL may influence how PSTs process information and manage academic demands, ultimately shaping their achievement levels^16–21^. Therefore, examining the relationship between GenAITU behaviors and AA, with mediating roles of COL and SMC, provides critical insights into how technology supports learning in teacher education to achieve SDGs. We operationalized AA for SDG4 through four indicators: (1) PSTs’ confidence in inclusive teaching, (2) integration of technology in pedagogy, (3) promotion of gender equality, and (4) support for lifelong learning and equitable opportunities in the context of teacher education^22,23^.

Furthermore, SMC refers to the collaborative regulation of thinking processes among learners, particularly in group or peer-supported learning contexts^8^. For PSTs, SMC plays a vital role in developing reflective practices, planning, monitoring, and evaluating learning tasks collectively^20^. As educational settings increasingly integrate digital tools like GenAITU, the dynamics of shared thinking and cognition, metacognition and motivation are evolving^24^. Based on these insights, we operationalized SMC in this study using four indicators: (1) collaborative reflection with AI tools, (2) shared problem-solving strategies supported by AI, (3) group regulation of tasks through AI, and (4) peer feedback on the use of AI for collaborative learning. Accordingly, we examined the mediating role of SMC in the relationship between GenAITU and AA within the context of teacher training.

Similarly, COL refers to the use of external tools or resources to reduce the mental burden associated with information processing and task completion^25^. In academic contexts, students often engage in COL by relying on technologies like GenAITU to store, retrieve, or generate content^26^. In this study, we operationalized COL through three indicators: (1) reliance on AI tools to store and retrieve information, (2) use of technology to manage routine tasks and reduce cognitive load, and (3) leveraging AI to enhance focus on higher-order thinking. We assumed that COL may serve as a mediating factor in the connection between GenAITU behaviors influence AA. This study, therefore, measured the direct and indirect effects of GenAITU on AA, with COL studied as a mediator variable.

**Fig. 1:**
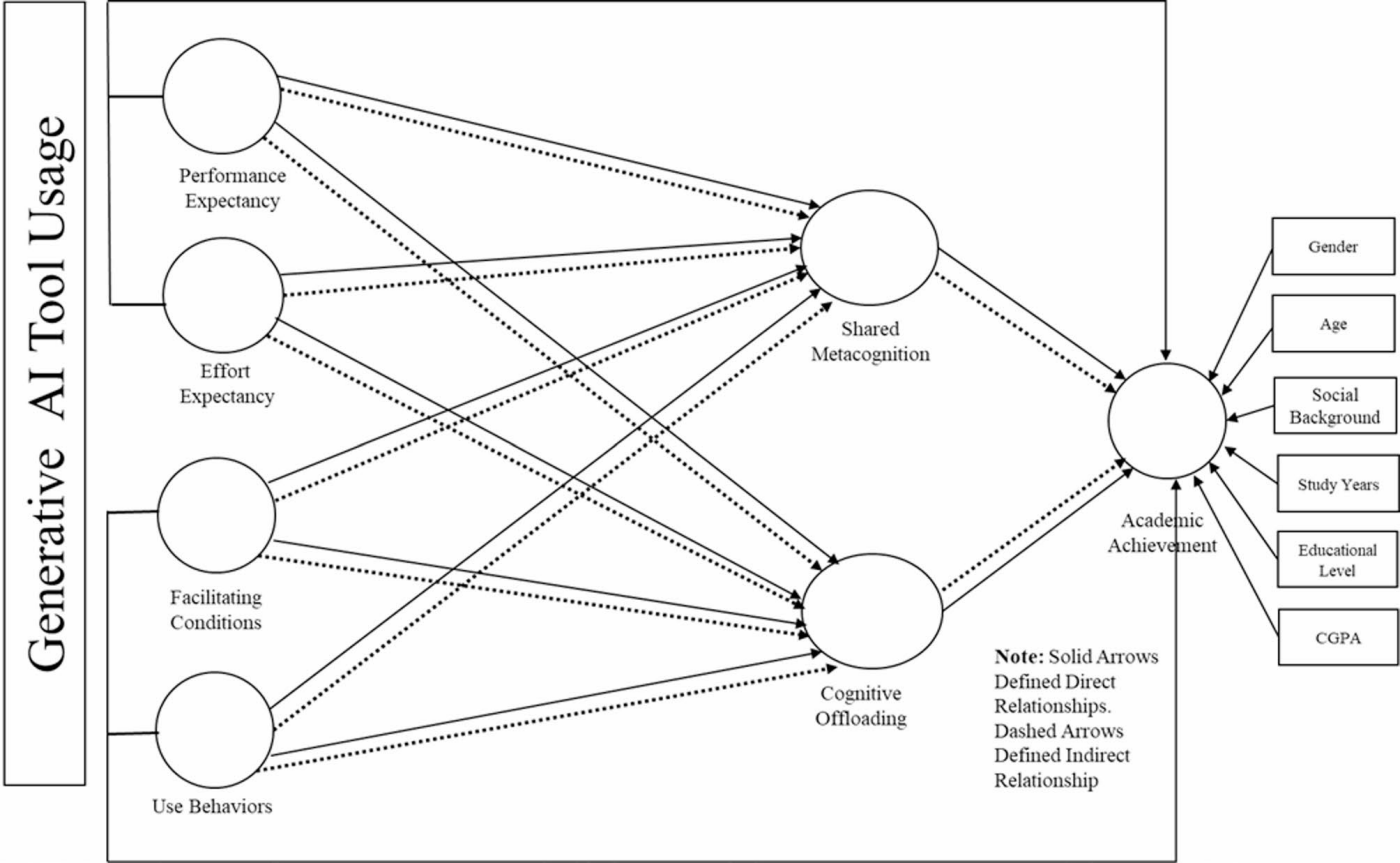
Proposed Research Model Illustrating Direct and Indirect Effects of GenAITU Subscales on AA via SMC and COL.

GenAITU behavior is a supportive tool to enhance learning in higher education^27^. It refers to the extent to which students engage with generative AI technologies, such as ChatGPT or other LLM-based tools, to support academic tasks. Consistent with UTAUT2, This study employs constructs from adapting UTAUT2 like PE, EE, FC, and UB to examine PSTs adoption behavior of generative AI tools for academic purposes^1,28^. Research indicated that GenAITU enhances academic growth to develop skills and knowledge about a sustainable future^3,4^. Similarly, research on generative AI has extended to areas such as mental health^29^, innovation management^30^, research agenda^31^, academic achievement^32^, academic research productivity^33^, entrepreneurial resilience^34^, teaching and learning^14^, and automating media^35^. This highlights the broadening role of generative AI across various fields. The authors note inconsistent findings regarding the impact of GenAITU on AA, highlighting the need for further research to clarify these effects. This study, therefore, addresses two key questions: (1) how GenAITU enhances PST’s AA in achieving SDG4 and (2) the mediating roles of SMC and COL in this process. Using the UTAUT2 model and SMC and COL perspectives, the study proposes that GenAITU increases SMC and COL to enhance PST’ AA toward SDG4 (see Fig. 1).

Our study fills various gaps in existing literature, particularly on how GenAITU influences the AA of achieving SDG4 among PST. This study also provides evidence on how GenAITU enhances knowledge and skills for fostering sustainable and inclusive education practices. The mediating roles of SMC and COL in the connection between GenAITU and AA were unexplored. This could be a limited understanding of how collaborative thinking and reduced cognitive load facilitated by GenAITU contribute to enhancing AA. Educational institutional management has valuable opportunities to integrate GenAITU which could improve teaching methodologies, curriculum development, and teacher preparedness for addressing global educational challenges. Our study also extends theoretical understanding by utilizing the UTAUT2 model to explore the effects of GenAITU on SMC and COL, enhancing knowledge and skills for achieving SDG4. For this purpose, we formulated the following research questions:

### Research question 1

How does GenAITU relate to AA among PSTs?

### Research question 2

How do SMC and COL relate to AA among PSTs?

### Research question 3

How do SMC and COL mediate the relationship between GenAITU and AA among PSTs?

Our study offers several contributions. First, it broadens the application of the UTAUT2 model by examining the effects of GenAITU on SMC, COL, and AA among PST. Second, it provides valuable insights for teacher education programs, highlighting how integrating GenAITU can enhance teacher training curricula and support SDG4 goals. Third, this research offers educational institutions strategies to innovate teaching methodologies and improve student outcomes through AI integration. Finally, the study bridges technology and education by promoting collaborative learning, reducing cognitive load, and fostering critical thinking, thereby supporting global educational objectives.

Our study begins with an introduction, followed by a literature review that includes the conceptual framework and hypothesis formulation. The methodology section then outlines the research design, data collection methods, and procedures. The next section presents the analysis, which discusses the findings. Finally, the discussion section interprets the results, provides conclusions, outlines implications, acknowledges limitations, and suggests areas for future research.

## Literature review

### Research model

This study draws upon three foundational theoretical frameworks to construct and analyze the proposed research model: the UTAUT2, the theory of SMC, and the COL theory (see Fig. 1). The UTAUT2 model^36^ provides the basis for understanding PSTs GenAITU behavior. Key constructs from UTAUT2 such as PE, PE, FC, and UB inform how GenAITU is likely to be adopted in educational settings. These elements are essential for understanding the behavioral drivers that influence how PSTs engage with AI tools in their academic work. UTAUT2 has been widely applied in educational technology research, making it a robust and reliable framework for analyzing AI technology use and acceptance behaviors in education contexts^10,37^.

In parallel, the theories of SMC^38^ and COL^16^ help explain the cognitive and collaborative processes involved when PSTs integrate GenAITU into their academic practices. SMC theory emphasizes the collective regulation of thinking and learning in group contexts, particularly how learners reflect, plan, and monitor tasks collaboratively. This is especially relevant in AI-supported learning environments, where interaction with both peers and technology influences learning outcomes. COL theory, on the other hand, highlights how individuals use external tools like generative AI tools to reduce mental effort by offloading memory, organization, and decision-making tasks. Together, these two theories support the hypothesized mediating roles of SMC and COL between GenAITU and AA. Finally, SDG4 as Quality Education is used as a guiding framework for conceptualizing AA, emphasizing inclusive, equitable, and innovative teaching strategies^22,23^. Indicators from SDG4 help anchor the outcome variable in globally recognized educational development goals^39^, ensuring that the study aligns with broader policy and pedagogical priorities. The following section deals with the operational definition of terms and hypotheses formulation.

### Generative artificial intelligence tool usage

GenAITU refers to students’ usage of generative AI technologies to support personalized learning, receive instant feedback, and access adaptive content^40^. Generative AI helps students simplify academic tasks, fostering collaboration and promoting critical thinking education. It enables deeper engagement with content by automating routine tasks and offering adaptive learning, contributing to enhanced AA, particularly in achieving SDG4,^3,4^. We adopted the GenAITU subscales, including PE, EE, FC, and UB from UTAUT2 ^10,40,41^. These four key factors were aligned with our study’s purpose to explore the connection between GenAITU on AA. In this study, GenAITU behavior is operationally defined as PSTs’ engagement with GenAITs for academic purposes influenced by PE, EE, FC, and UB from UTAUT2, in relation to enhancing AA aligned with SDG4.

### Shared metacognition

SMC refers to the collaborative regulation of cognitive tasks, where learners collectively reflect, monitor, and adjust their learning strategies^42,43^. This concept is drawn from collaborative learning theory. It serves as a mediator in our research model, showing how GenAITU facilitates group learning, reflection, and improved AA^42,43^. In this study, we introduce SMC as PSTs’ ability to engage in group problem-solving and coordinate academic tasks, with AI aiding in structuring discussions, tracking contributions, and fostering reflection. In this study, we operationalized shared metacognition using four indicators: (1) collaborative reflection with AI tools, (2) shared problem-solving strategies supported by AI, (3) group regulation of tasks through AI, and (4) peer feedback on the GenAITU for collaborative learning. This collaborative approach enhances learning and supports critical decision-making, essential for improving AA and advancing SDG4 ^3,40,41^.

### Cognitive offloading

COL involves delegating cognitive tasks, like information retrieval or memory management, to external tools, reducing mental workload. In this study, generative AI tools enable PST to offload simpler tasks, allowing them to focus on complex academic challenges^19^. By automating routine tasks, AI reduces cognitive strain, freeing up mental resources for higher-order thinking and problem-solving^44^. This offloading supports more efficient learning and enhances AA by helping students manage cognitive load and excel, particularly in sustainable education contexts. Based on COL theory, the research model used COL as a mediator, showing how GenAITU helps PST manage cognitive demands. By automating tasks, cognitive resources are better allocated, improving AA^18,25^.

### Academic achievement

AA refers to measurable learning outcomes such as self-reported perceived learning gains, reflecting students’ academic success^2^. In the context of this study, AA is influenced by the integration of GenAITU, which enhances learning by offering personalized support, fostering collaboration through SMC, and reducing mental effort via COL. These mechanisms help PSTs engage more deeply with academic content, promote critical thinking, and ultimately improve learning outcomes. This framework aligns with the broader objectives (SDG4), ensuring inclusive, equitable, and quality education for all, along with promoting lifelong learning opportunities^3,4^. In this research, we operationalized AA for SDG4 through four indicators: (1) PSTs’ confidence in inclusive teaching, (2) integration of technology in pedagogy, (3) promotion of gender equality, and (4) support for lifelong learning and equitable opportunities in teacher education. Hence, the study examines how GenAITU, mediated by SMC and COL, contributes to improved AA, supporting calls for transformative digital pedagogies in higher education^39,41^.

## Hypotheses formulation

### **Generative artificial intelligence tool usage vs. Academic achievement**

Several studies indicated that GenAITU has become a transformative tool for fostering AA^40,45^. Torres^46^ found that GenAITU engages students more in complex topics and fosters AA. Consequently, it has become vital to sustainable academic success^47^. Yusuf, et al.^48^ demonstrated that structured GenAITU, when guided by a critical thinking framework, can enhance academic achievement by improving students’ ability to evaluate and synthesize AI-generated content. A recent meta-analysis by Dong, et al.^49^ confirmed that AI integration has a significant positive effect on students’ academic achievement, with a large effect size (0.924), highlighting its transformative potential in education. Sun and Zhou^32^ found that GenAITU significantly enhances college students’ academic achievement, particularly when used in independent learning contexts and for generating textual content. In the context of PST aiming to achieve SDG4, the role of generative AI becomes even more significant in addressing academic challenges. To the best of the authors’ knowledge, there is a lack of empirical evidence in existing literature. Therefore, we introduce the exploration of connections between PE, EE, facilitating condition, and UB as part of GenAITU, and AA, as examined in the following hypotheses:

*H1a: PE positively contributes to AA.*

*H1b: EE positively contributes to AA.*

*H1c: FC positively contributes to AA.*

*H1d: UB positively contributes to AA.*

### Generative artificial intelligence tool usage vs. Shared metacognition

Similarly, several studies have emphasized the connection between GenAITU and SMC, particularly in how students collaborate and regulate their cognitive tasks^13,50^. For example, researchers also highlighted that GenAITU enhances the collective regulation of thinking processes among student groups^51^. Another study found that this tool enhances individual creativity and reduces the diversity of novel content at the collective level. With AI-driven collaboration platforms, students can engage in joint problem-solving, reflect on contributions, and collectively adjust their learning strategies^51^. Another study indicated that personality traits influence learners to use generative AI like ChatGPT for SMC^52^. To the authors’ knowledge, these insights have yet to be explored in the context of PST working toward SDG4. Therefore, this study examines the relationship between GenAITU (PE, EE, FC, and UB) and SMC, as outlined in the following hypotheses:

*H2a: PE positively contributes to SMC.*

*H2b: EE positively contributes to SMC.*

*H2c: FC positively contributes to SMC.*

*H2d: UB positively contributes to SMC.*

### Generative artificial intelligence usage vs. Cognitive offloading

Several studies have investigated the connection between GenAITU and COL across various contexts^26,53^. For example, technologies-enabled job crafting illustrates how AI tools facilitate task customization and innovation, thereby reshaping job roles, improving employee autonomy, and enabling COL^44^. Research has also shown that optimizing input and feedback methods on mobile touch devices can significantly enhance COL in elderly users, with the stylus and combined audio-visual feedback being the most effective in reducing cognitive load and increasing user satisfaction^54^. For PST focused on SDG4, understanding how generative AI supports COL can enhance teaching practices and curriculum design. Empirical evidence on GenAITU connection with COL among PST is currently limited. Thus, this study investigates the relationship between GenAITU (PE, EE, FC, and UB) and COL, as outlined in the following hypotheses:

*H3a: PE positively contributes to COL.*

*H3b: EE positively contributes to COL.*

*H3c: FC positively contributes to COL.*

*H3d: UB positively contributes to COL.*

### Shared metacognition vs. academic achievement

Recent research has shown that SMC plays a significant role in academia^55^. For example, the study highlighted the integration of SMC in learning, emphasizing how metacognitive practices can promote social justice advocacy and collaborative leadership^20^. The research also emphasized the importance of SMC in enhancing collaborative problem-solving in virtual laboratory settings, using learning analytics to reveal patterns and their impact on problem-solving outcomes^55^. Another study highlighted that stronger engagement in socially shared regulation of learning and positive academic emotions significantly boost academic performance^56^. In the context of PST working towards SDG4, SMC can support collaborative teaching practices and foster critical thinking skills. All these insights align with our study on how SMC enhances AA, though this relationship remained unexplored. Therefore, we investigate how SMC positively contributes to AA, as examined in the following hypothesis:

*H4: SMC positively contributes to AA.*

### **Cognitive offloading vs. academic achievement**

COL enhances AA by allowing students to delegate tasks to external tools or technologies, thereby enabling them to focus on higher-order thinking and problem-solving^25^. Offloading irrelevant information to external tools has been shown to improve cognitive performance in unrelated tasks by freeing up mental resources^17^. However, a reduction in offloading has been associated with a decline in immediate task performance, emphasizing the balance between short-term and long-term cognitive gains^17^. Additionally, Weis and Wiese^53^ found that COL is flexible, adjusting based on performance goals—participants prioritize speed or accuracy depending on task objectives, illustrating how humans adapt offloading strategies to optimize performance. As per the authors’ understanding, there is a lack of empirical research exploring how COL contributes positively to AA. Thus, this study investigates this relationship through the following hypothesis:

*H5: COL contributes to AA.*

### An integrative mediation model

In summary, hypotheses H1.1-H5 propose that GenAITU contributes to AA, enhances SMC, and facilitates COL. Both SMC and COL positively influence PST’ AA. These relationships form the basis of the hypotheses presented in Fig. 1. We further hypothesize that GenAITU influences AA through SMC (H6.1-H6.4). Similarly, GenAITU impacts AA through COL (H7.1-H7.4). Overall, these hypotheses suggest a mediation model (see Fig. 1). Taken together, we propose the following mediating hypotheses:

*H6.1: SMC mediates the relationship between PE and AA.*

*H6.2: SMC mediates the relationship between EE and AA.*

*H6.3: SMC mediates the relationship between FC and AA.*

*H6.4: SMC mediates the relationship between UB and AA.*

*H7.1: COL mediates the relationship between PE and AA.*

*H7.2: COL mediates the relationship between EE and AA.*

*H7.3: COL mediates the relationship between FC and AA.*

*H7.4: COL mediates the relationship between UB and AA.*

## Methods

### Research design and participants

**Fig. 2:**
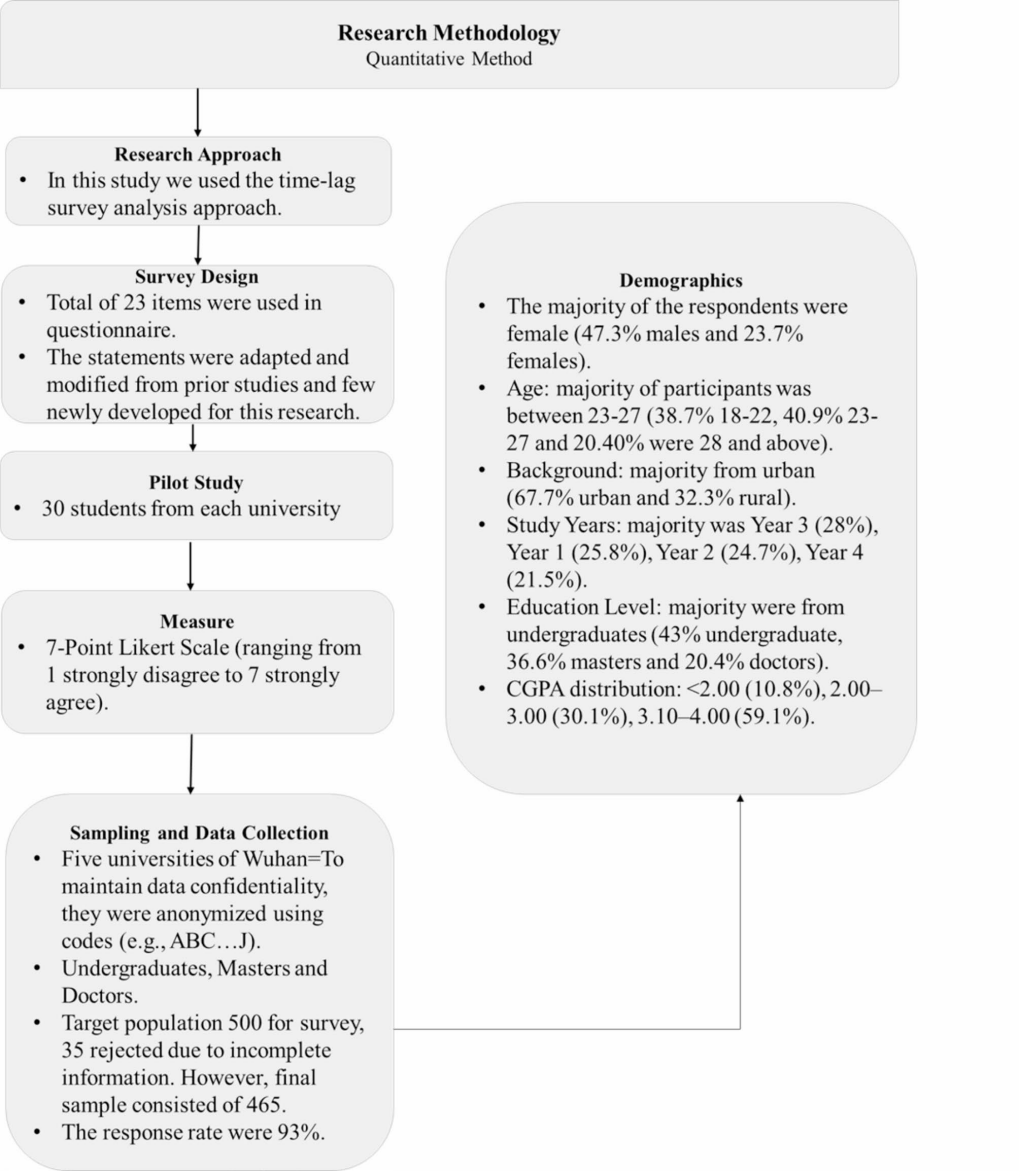
Methodology flowchart providing an overview of the research methodology and demographic characteristics of the participants. The left panel outlines the stepwise research methodology, employing a time-lagged quantitative survey approach. The right panel summarizes key demographic details of the sample.

This study employed a quantitative, time-lag survey design to examine the relationship between GenAITU, SMC, COL, and AA among PST in the context of SDG4. Participants were conveniently selected from bachelor’s, master’s, and doctoral students across five universities in Wuhan, China, who had completed an SDG4-related course and used generative AI tools. A total of 465 usable responses were collected out of 500 targeted participants (93% response rate) during March-May 2024 through structured, online surveys via WeChat QR codes. Participation was voluntary and anonymous, with consent obtained from both university management and participants. Surveys were conducted in two phases, with 230 responses at Time 1 and 235 follow-ups at Time 2 (2 weeks apart). The sample included 47.3% males and 52.7% females, predominantly aged 18–27 years (79.6%), with urban backgrounds (67.7%). Most were undergraduates (43%), with CGPAs ranging between 3.10 and 4.00 (59.1%) (Table 1; Fig. 2).

### Ethics approval and consent to participate

The study received ethical approval for a survey from Institutional Ethical Review Boards (IRB), XXX (Approval No. IERB-SED/2305/2023), approved on May 23, 2023. In addition to the approval of the study, informed consent was obtained from all participants, who were fully informed about the study’s purpose, procedures, and their rights, including the voluntary nature of participation and the option to withdraw at any time without penalty. This study did not involve experiments on humans or the use of human tissue samples, instead, it involved a survey. All procedures complied with the ethical principles outlined in the Declaration of Helsinki.

**Table 1:** Demographic characteristics of the study participants (*N* = 465). The table presents the frequency and percentage distribution of respondents based on gender, age group, residential background, year of study, education level, and cumulative grade point average (CGPA). The sample includes undergraduate, master’s, and doctoral students from five universities.

Variable	Category	Frequency (f)	Percentage
Gender	Male	220	47.3%
	Female	245	52.7%
	Total	465	100%
Age	18–22 years	180	38.7%
	23–27	190	40.9%
	28 and above	95	20.40
	Total	465	100%
Background	Rural	150	32.3%
	Urban	315	67.7%
	Total	465	100%
Study Years	Year 1	120	25.8%
	Year 2	115	24.7%
	Year 3	130	28.0%
	Year 4	100	21.5%
	Total	465	100%
Education Level	Undergraduate	200	43.0%
	Masters	170	36.6%
	Doctoral	95	20.4%
	Total	465	100%
CGPA	Less than 2.00	50	10.8%
	2.00–3.00	140	30.1%
	3.10-4.00	275	59.1%
	Total	465	100%

### Measures

All scales were adapted from previous studies, with their validity and reliability ensured (see Table 2):

PE (4 items, α = 0.791), e.g., “I believe that AI Generative tool is useful in my studies”^10^.

EE (3 items, α = 0.701), e.g., “Learning how to use AI Generative tool is easy for me”^10^.

FC (3 items, α = 0.759), e.g., “I have the resources necessary to use AI Generative tool”^10^.

UB (4 items, α = 0.786), e.g., “I use AI Generative tools to complete my assignments and projects”^10^.

SMC (5 items, α = 0.777), e.g., “I collaborate with my peers to reflect on the progress of our learning tasks when using AI tools”^42,43^.

COL (4 items, α = 0.766), e.g., “AI systems enable me to offload mental tasks that would otherwise occupy my memory.”^18,25^.

AA (6 items, α = 0.800), e.g., “I understand the importance of gender equality in education and feel prepared to promote it in my teaching practices”^39,41^.

All items were rated on a 7-point Likert scale. Gender, age, background, field of study, educational level, and CGPA were control variables.

### Data analysis procedure

The data analysis involved both descriptive and inferential statistics. Descriptive statistics summarized the demographic characteristics, while PLS-SEM was conducted in two phases: the measurement model phase assessed reliability, validity, and multicollinearity using Cronbach’s Alpha, Composite Reliability, indicator loadings, Average Variance Extracted, and Variance Inflation Factor. Following this, structural relationships among variables in the research model were evaluated using PLS-SEM.

## Results

A PLS-SEM approach (using SmarPLS 4) was employed to analyze the relationships in the theoretical framework. The variance-based SEM approach was selected due to its relatively less sensitivity to sample size compared to covariance-based SEM approaches such as AMOS. First, the reliability and validity of the scales were assessed^57^. Table 2 presents the reliability and validity results for all constructs, showing that reliability measures (Cronbach’s alpha, rho_A, and composite reliability) exceeded the 0.7 threshold, with each construct’s Average Variance Extracted above 0.5. Reliability, validity, and multicollinearity were further assessed using Cronbach’s Alpha, Composite Reliability, indicator loading, AVE, and Variance Inflation Factor.

All constructs demonstrated good internal consistency with Cronbach’s Alpha values, Composite Reliability (rho_a and rho_c) values above 0.70, and indicator loading above 0.6, indicating strong reliability. The AVE values for each construct were above the threshold of 0.50, confirming convergent validity, while the VIF values were all below 3, suggesting that multicollinearity was not a concern in this model (see Table 2).

**Table 2:** Reliability, convergent validity, and multicollinearity statistics of the measurement items. The table presents indicator loadings, variance inflation factors (VIF), Cronbach’s alpha (CA), composite reliability (CR) using both Rho_A and Rho_C, and average variance extracted (AVE). All measurement items demonstrate acceptable levels of internal consistency, convergent validity, and no significant multicollinearity issues.

Variables	Items	Indicator Loading	VIF
PE CA: 0.791 CR (rho_a): 0.797 CR (rho_c): 0.812 AVE: 0.523	PE1	0.645	1.217
	PE2	0.845	1.636
	PE3	0.745	1.430
	PE4	0.638	1.233
EE CA: 0.701 CR (rho_a): 0.707 CR (rho_c): 0.790 AVE: 0.557	EE1	0.707	1.179
	EE2	0.733	1.190
	EE3	0.796	1.267
FC CA: 0.759 CR (rho_a): 0.764 CR (rho_c): 0.814 AVE: 0.593	FC1	0.718	1.303
	FC2	0.781	1.249
	FC3	0.809	1.520
UB CA: 0.786 CR (rho_a): 0.789 CR (rho_c): 0.810 AVE: 0.517	UB1	0.756	1.431
	UB2	0.642	1.162
	UB3	0.704	1.271
	UB4	0.768	1.427
SMC CA: 0.777 CR (rho_a): 0.795 CR (rho_c): 0.847 AVE: 0.528	SM1	0.799	2.059
	SM2	0.653	1.434
	SM3	0.802	2.088
	SM4	0.706	1.406
	SM5	0.660	1.481
COL CA: 0.766 CR (rho_a): 0.779 CR (rho_c): 0.852 AVE: 0.592	CO1	0.797	1.591
	CO2	0.797	1.698
	CO3	0.633	1.236
	CO4	0.836	1.794
AA CA: 0.800 CR (rho_a): 0.803 CR (rho_c): 0.857 AVE: 0.557	AA1	0.770	1.640
	AA2	0.700	1.495
	AA3	0.658	1.320
	AA4	0.668	1.397
	AA5	0.706	1.582
	AA6	0.736	1.662

Cronbach’s alpha (CA), Composite reliability (CR) (rho_a),

Composite reliability (CR)(rho_c), Average variance extracted (AVE).

Henseler et al. (2015) criticized Fornell and Larcker’s approach to assess the discriminant validity in reflective scales, advocating for theheterotrait–monotrait (HTMT) method as a more appropriate alternative. Accordingly, we used the HTMT approach to measure discriminant validity, ensuring that none of the HTMT values exceeded the 0.90 threshold, thereby confirming that the constructs are distinct (Asghar, Arif, et al., 2021; Iqbal et al., 2021; Malik et al., 2021). Table 3 shows that all constructs had HTMT values below 0.90, supporting adequate discriminant validity.

**Table 3:** Discriminant validity assessment using the Heterotrait-Monotrait ratio (HTMT). The table presents the HTMT values between latent constructs to evaluate discriminant validity. Values below the recommended threshold of 0.85 indicate adequate discriminant validity among constructs.

Variables	AA	COL	EE	FC	PE	SMC	UB
AA							
COL	0.672						
EE	0.554	0.602					
FC	0.449	0.675	0.492				
PE	0.510	0.545	0.638	0.562			
SMC	0.537	0.433	0.709	0.484	0.401		
UB	0.533	0.451	0.430	0.407	0.435	0.406	

The model fit indices suggest a good fit for both the Saturated Model and the Estimated Model, as the SRMR values (0.069 and 0.070) are well within acceptable limits. This indicated that the models are well-specified. The d_ULS and d_G values are close, further supporting model stability. Notably, the NFI values (0.989 for the Saturated Model and 0.985 for the Estimated Model) are high, suggesting an excellent model fit relative to the null model, indicating that the models represent the data well.

The R-square values indicate that the model explains 40.8% of the variance in AA, 34.5% in COL, and 30.7% in SMC. The adjusted R-square values, slightly lower, suggest a small reduction in explanatory power due to the inclusion of additional predictors, indicating that the model remains robust in predicting these outcomes.

The f-square values provide insights into the effect sizes of the variables in the model. For example, the effect size of SMC on AA is 0.049, suggesting a small but notable impact. Meanwhile, COL has a slightly larger effect size on AA with an f-square of 0.127, indicating a more substantial contribution to the variance in AA. Overall, the variables display varying levels of impact, with COL showing a relatively stronger influence compared to other predictors.

### Structural modeling

We measured the direct effect of GenAITU (PE, EE, FC, and UB) on AA. The results indicated that GenAITU subscales like PE have a positive and significant relationship with AA (β = 0.130, *p* < 0.05), which approved our hypothesis H1.1. However, EE and FC have no positive and significant connection with AA (β = 0.031, *p* > 0.05; β = -0.005, *p* > 0.05), which does not support our hypotheses H1.2 and H1.3. The analysis results show that PE significantly impacts AA (β = 0.130, *p* < 0.05), which approved hypothesis H1.4.

Our study also measured the direct effect of GenAITU (PE, EE, FC, and UB) on SMC. The results indicated that GenAITU subscales like PE do not have a positive and significant relationship with SMC (β = 0.019, *p* > 0.05), which does not approve our hypothesis H1.1. However, EE, FC, and UB have a positive and significant connection with SMC (β = 0.396, *p* < 0.05; β = 0.191, *p* < 05; β = 0.124, *p* < 0.05), which supported our hypotheses H2.2, H2.3 and H2.4, respectively.

This study also measured the direct effect of GenAITU (PE, EE, FC, and UB) on COL. The results indicated that GenAITU subscales PE, EE, FC, and UB have positive and significant connections with COL (β = 0.139, *p* < 0.05; β = 0. 214, *p* < 05; β = 0. 327, *p* < 0.05; β = 0. 132, *p* < 0.05), which approved our hypotheses H3.1, H3.2, H3.3 and H3.4, respectively.

**Fig. 3:**
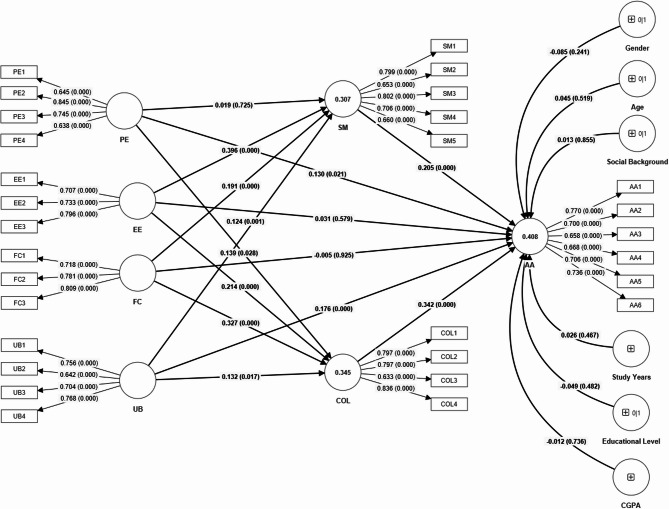
Structural model illustrating the direct and indirect relationships among the study constructs. The model examines the influence of Performance Expectancy (PE), Effort Expectancy (EE), Facilitating Conditions (FC), and Use Behavior (UB) on Shared Metacognition (SM) and Cognitive Offloading (COL), which in turn impact Academic Achievement (AA). Path coefficients and p-values are displayed along the arrows. Outer loadings for individual items and R² values for endogenous constructs are also presented. Demographic variables (gender, age, social background, study years, educational level, and field of study) were tested as control variables and found to have no significant effects on AA.

Finally, both SMC and COL directly, positively, and significantly influence AA (β = 0.205, *p* < 0.05; β = 0.342, *p* < 0.05). Thus, H4 and H5 are accepted. Control variables such as gender, age, educational level, study years, educational level, cumulative grade point average (CGPA) did not have a significant effect on AA (β = -0.085, *p* > 0.05; (β = 0.045, *p* > 0.05; (β = 0.013, *p* > 0.05; (β = 0.026, *p* > 0.05; (β = -0.049, *p* > 0.05; (β = -0.012, *p* > 0.05) (See Table 4; Fig. 3).

**Table 4:** Direct effects of technology-related constructs, shared metacognition, cognitive offloading, and control variables on academic achievement. The table reports path coefficients, mean estimates, standard deviations (SD), t-values, and p-values for each hypothesized relationship. Statistically significant results are indicated by ***. Control variables such as gender, age, social background, year of study, educational level, and CGPA were included to account for their potential influence on academic outcomes.

Hypotheses	Direct Relations	Coefficients	Mean	SD	T Value	*P* values	Decisions
H1.1	PE -> AA	0.130	0.132	0.056	2.302	0.021	***Sig
H1.2	EE -> AA	0.031	0.031	0.056	0.555	0.579	Not***Sig
H1.3	FC -> AA	-0.005	-0.006	0.057	0.094	0.925	Not***Sig
H1.4	UB -> AA	0.176	0.178	0.04	4.406	0.000	***Sig
H2.1	PE -> SMC	0.019	0.019	0.053	0.352	0.725	Not***Sig
H2.2	EE -> SMC	0.396	0.398	0.049	8.096	0.000	***Sig
H2.3	FC -> SMC	0.191	0.19	0.051	3.744	0.000	***Sig
H2.4	UB -> SMC	0.124	0.127	0.038	3.254	0.001	***Sig
H3.1	PE -> COL	0.139	0.143	0.063	2.205	0.028	***Sig
H3.2	EE -> COL	0.214	0.209	0.061	3.541	0.000	***Sig
H3.3	FC -> COL	0.327	0.327	0.054	6.015	0.000	***Sig
H3.4	UB -> COL	0.132	0.135	0.055	2.393	0.017	***Sig
H4	SMC -> AA	0.205	0.205	0.055	3.759	0.000	***Sig
H5	COL -> AA	0.342	0.343	0.057	5.982	0.000	***Sig
Control Variables	Gender -> AA	-0.085	-0.084	0.072	1.173	0.241	Not***Sig
	Age -> AA	0.045	0.044	0.07	0.645	0.519	Not***Sig
	Social Background -> AA	0.013	0.012	0.071	0.183	0.855	Not***Sig
	Study Years -> AA	0.026	0.026	0.035	0.728	0.467	Not***Sig
	Educational Level -> AA	-0.049	-0.047	0.07	0.703	0.482	Not***Sig
	CGPA -> AA	-0.012	-0.012	0.036	0.337	0.736	Not***Sig

We further measured the mediating roles of SMC and COL in the relationship between GenAITU (PE, EE, FC, and UB) and AA. The results indicated that PE does not indirectly affect AA through SMC (β = 0.004, *p* > 0.05), which does not support our hypothesis H6.1. However, EE, FC, and UB have an indirect, positive, and significant effect on AA through SMC (β = 0.081, *p* < 0.05; β = 0.039, *p* < 0.05; β = 0.045, *p* < 0.05), which approved our hypothesis H6.2 H6.3 and H6.4. The results also indicated that GenAITU subscales like PE, EE, FC, and UB have an indirect, positive, and significant relationship on AA through COL (β = 0.048, *p* < 0.05; β = 0.073, *p* < 0.05; β = 0.112, *p* < 0.05; β = 0.025, *p* < 0.05), which approved our hypotheses H7.1, H7.2, H7.3, and H7.4 (See Table 5; Fig. 3).

The type of mediation demonstrated in the results is partial mediation. This is evident as both direct and indirect effects of variables such as EE, FC, and UB are significant in influencing AA through mediators like SMC and COL. This suggests that the mediators partially explain the relationships.

**Table 5:** Indirect relationships between GenAITU subscales and AT through SMC and COL. The table presents the coefficients, means, standard deviations, t-values, and p-values for each hypothesis. Significant relationships (*p* < 0.05) are indicated as ***Sig.

Hypotheses	In-direct Relations	Coefficients	Mean	SD	T Value	*P* values	Decisions
H6.1	PE -> SMC -> AT	0.004	0.004	0.011	0.335	0.738	Not***Sig
H6.2	EE -> SMC -> AT	0.081	0.081	0.022	3.717	0.000	***Sig
H6.3	FC -> SMC -> AT	0.039	0.039	0.014	2.753	0.006	***Sig
H6.4	UB -> SMC -> AT	0.045	0.047	0.022	2.09	0.037	***Sig
H7.1	PE -> COL -> AT	0.048	0.048	0.021	2.266	0.023	***Sig
H7.2	EE -> COL -> AT	0.073	0.073	0.028	2.661	0.008	***Sig
H7.3	FC -> COL -> AT	0.112	0.111	0.025	4.473	0.000	***Sig
H7.4	UB -> COL -> AT	0.025	0.026	0.011	2.323	0.020	***Sig

## Discussion

Our study expands the understanding of PST’ perceptions of how GenAITU influences AA to achieve SDG4. Given the limited research in this area, especially within higher education, our findings are vital for advancing discussions on the role of GenAITU in academic settings. This study adapted the UTAUT 2 model to assess GenAITU, using its four external factors like PE, EE, FC, and UB. Other variables were excluded because focusing on these four key factors aligned with our aims of exploring GenAITU impact on AA. These variables provided a solid foundation for analyzing PST’ GenAITU. Including additional variables could have overcomplicated the study without yielding significant new insights.

Our study confirmed that two out of four UTAUT2 constructs, such as PE and UB as part of GenAITU positively and significantly contribute to AA, thus supporting hypotheses H1.1 and H1.4. These findings are consistent with previous research that has demonstrated the positive impact of PE and UB on academic outcomes in higher education, as reported in earlier studies on the UTAUT2 model, particularly with regard to PE’s effect on academic performance^58^. On the other hand, EE and FC as part of GenAITU did not significantly influence AA, which led to the lack of support for our hypotheses H1.2 and H1.3. These results were inconsistent with findings reported in prior research, which suggested that EE and FC play a significant role in enhancing performance outcomes in AI-driven academic contexts^59^. However, our findings align with studies in technology adoption literature where EE and FC failed to predict outcomes^60,61^. Such results may have emerged due to PST’ varying levels of digital literacy and the specific challenges they face in aligning GenAITU with the pedagogical demands of SDG4, the assumptions on EE and FC are required more investigation among PST’ perceptions.

Furthermore, our results indicated that PE as part of GenAITU did not contribute to SMC, which did not support our hypothesis H2.1. The results were inconsistent with findings reported by previous research, which demonstrated that PE is often a significant predictor of socially shared metacognitive engagement, particularly when AI tools are used for collaborative learning and problem-solving tasks^62^. Similarly, another research indicates that perceived usefulness, a component of performance expectancy, significantly affects the use of artificial intelligence in education, which in turn positively impacts learning performance^63^. It can be assumed that the UTAUT2 component PE, in the context of measuring GenAITU behavior, requires further investigation regarding its impact on AA from the perspective of PSTs in pedagogical settings. On the other hand, EE, FC, and UB positively and significantly contribute to SMC, which supports our hypotheses H2.2-H2.4. These results are consistent with previous studies highlighting the significant influence of EE and FC on behavioral intentions and learning engagement, particularly in technology-driven educational settings^37,64^. In addition to our findings, a study highlighted the potential of generative AI in enhancing students’ learning experiences and aiding teachers in delivering better educational outcomes^65^. The study also demonstrated that AI-supported instruction allows for customized learning experiences^9^. These results may reflect the PST’ limited familiarity with GenAITU (PE) collaborative learning potential.

Additionally, our outcomes indicated that PE, EE, FC, and UB as part of GenAITU positively and significantly contributed to COL, which supports H3.1-H3.4. The results were consistent with previous studies that highlighted the significant role these factors play in enhancing the adoption and integration of AI tools, particularly in educational settings where cognitive load management is important^66^. Previous research has also found that PE and FC help students offload cognitive tasks^26^. Another study indicated that ICT-enabled job crafting demonstrates how AI tools support task customization and innovation and enhance COL^44^. These results likely occurred due to GenAITU simplifying complex tasks and enhancing COL in the context of preservice teacher education to achieve SDG4.

Our study indicated that SMC contributed positively and significantly to AA, which approved our hypothesis H4. The results align with previous research showing that SMC enhances academic outcomes and performance through collaborative thinking in small groups among pharmacy graduates^8^. Singh and Muis^67^ found a correlation between SMC and motivation toward learning in a preservice teaching program. Iiskala, et al.^38^ found that SMC was more frequent during difficult problems, facilitating problem-solving through collaboration. Sulla, et al.^68^ emphasized teachers’ strategies, such as enhancing students’ socially shared regulatory strategies for learning. This highlights the importance of fostering SMC in preservice teacher education to support the achievement of SDG4.

Likewise, results indicated that COL positively and significantly contributed to AA, which supported hypothesis H5. The results were consistent with the previous studies reported by Berry, et al.^69^ that COL improves task performance among students with low memory. Grinschgl, et al.^25^ found that increased COL improves secondary task performance in demanding multitasking situations, which is also aligned with our results. Morrison and Richmond^19^ found that COL benefits short-term memory performance, especially at higher memory loads. These findings highlight the potential of COL as a valuable strategy to enhance the learning experiences of PST, aligning with SDG4.

Our study revealed that SMC mediated the relationship between GenAITU (EE, FC, and UB) and AA, supporting hypotheses H6.2 and H6.4, while PE had no indirect effect, disproving hypothesis H6.1. Dahri, et al.^70^ explored how AI-generative tools found that FC positively impacts the acceptance and effective use of AI, enhancing academic outcomes. Lobczowski, et al.^8^ ) found that SMC enhanced project-based learning among pharmacy graduate students, while Biasutti and Frate^42^ demonstrated that SMC in online collaborative learning improved outcomes among university students. Together, these findings highlight how SMC strengthens the relationship between AA, especially for PST, supporting the acquiring the knowledge of SDG4.

Our study revealed that COL mediated the relationship between GenAITU (PE, EE, FC, and UB) and AA, supporting hypotheses H7.1 and H7.4. Previous studies have shown that generative AI tools decrease cognitive load management^66^, while PE and FC help students offload cognitive tasks^26^. Similarly, ICT-enabled support facilitates task customization innovation and enhances COL^44^. Altogether, these findings indicate that GenAITU enhances AA through COL, particularly in the context of preservice teacher education, aligning with the goals of SDG4.

## Conclusions

Our synthesized research framework was constructed based on the UTAUT2 model, socially shared regulation, and cognitive overload theory, incorporating insights from prior literature to examine the hypothesized relationships among the key variables. The results confirmed the direct and indirect relationship of GenAITU with AA among PST in the context of SDG4 in China. The study revealed that two GenAITU subscales (PE and UB) directly, positively, and significantly affect AA. The two subscales of GenAITU (EE and FC) have no direct and significant effect on AA.

We also measured the direct connection of GenAITU with SMC, and the results indicated that one subscale of GenAITU (PE) has no direct and significant connection with SMC. The three subscales of GenAITU (EE, FC, and UB) directly, positively, and significantly affect SMC. This study also measured the direct effect of GenAITU on COL. The results indicated that all subscales of GenAITU (PE, EE, facilitating conditioning, and UB) directly, positively, and significantly affect COL. We also measured the direct effect of SMC on AA. Results indicated that SMC has a direct, positive, and significant effect on AA. We also measured the direct effect of COL on AA. Results indicated that COL has a direct, positive, and significant effect on AA.

This study also measures the indirect relationship of GenAITU with AA through SMC and COL. The results indicated that GenAITU (EE, FC, and UB) directly affects AA through SMC. In contrast, PE does not affect it. The results also indicated that GenAITU (PE, EE, FC, and UB) indirectly affects AA through COL.

This study can draw the following conclusions: First, PE and UB are key predictors of AA among PST, indicating that the belief in technology improving performance and its actual usage significantly impact their academic progress. Second, EE, FC, and UB are crucial factors that significantly enhance SMC. Third, PE, EE, FC, and UB are essential factors that significantly improve COL. Fourth, SMC can predict AA. Fifth, COL is also a key factor in enhancing AA. Sixth, socially shared metacognitive strategies are effective in making GenAITU beneficial for enhancing AA among PST in the context of SDG4. Seventh, COL supports and enhances the effectiveness of GenAITU in improving AA among PST in the context of SDG4.

### Implications

The usage of generative AI-SMC and COL as key predictors of AA in the context of SDG4 highlights actionable strategies for stakeholders. For teachers, training programs should adopt pedagogical frameworks like the SUBtitution–Augmentation–Modification–Redefinition (SAMR) model to scaffold AI integration, for example, using ChatGPT to augment peer feedback or redefine collaborative tasks. Micro-credential modules could equip educators to guide students in critically evaluating AI outputs. Curriculum policymakers should embed AI literacy into standards using Technological Pedagogical Content Knowledge (TPACK), such as mandatory courses on “AI for SMC and COL” that teach goal setting via AI-generated study plans. Discipline-specific guidelines and tiered AI Resource HUB can address skill gaps equitably. University management must prioritize infrastructure, such as Generative AI Sandbox Environments for experimentation with GenAI tools, paired with analytics to refine support for faculty and students. Clear governance frameworks should mandate ethical AI use declarations in assignments and train faculty on AI plagiarism detection tools. For pre-service teachers (PSTs), pedagogy labs could simulate AI-driven classroom scenarios, while mentorship programs co-design AI-enhanced lesson plans for metacognitive development. These strategies ensure AI integration aligns with pedagogical goals and institutional readiness, fostering SMC, COL, and AP which are critical to SDG4.

### Limitations and future research

Our study also has some limitations that should be considered when the results are interpreted. First, the sample for this study was limited to China, where access to some renowned AI tools is restricted due to government regulations. This may affect the generalizability of the findings to students in other contexts. Additionally, the reliance on self-reported data introduces potential biases, as participants may have been influenced by social desirability or inaccuracies in recalling their experiences with generative AI technologies. While access to behavioral or observational data (e.g., AI system usage logs or analytics) was not feasible within the scope and timeframe of this study, such triangulation could have strengthened the validity of the findings. We recommend that future research incorporate these behavioral data sources to complement self-reports and enhance the robustness of the results. Furthermore, the time-lag design of the study prevents an examination of how students’ perceptions might evolve as their exposure to and experiences with generative AI technologies increase. Furthermore, while the time-lagged design improves upon cross-sectional methods by introducing temporal separation between variables, it does not fully establish causal relationships, a limitation that also applies to the mediation analysis conducted in this study, as causality is a key assumption in mediation. Additionally, the time-lag design prevents an examination of how students’ perceptions might evolve over extended periods as their exposure to and experiences with generative AI technologies increase.

Moreover, because generative AI has not been fully integrated into formal academic settings, students had limited exposure to it. This study did not explore how students from different fields, such as science, technology, engineering, mathematics, or medicine, were exposed to generative AI and the actual impact on their learning outcomes. This would be essential for a more comprehensive understanding of the role of these technologies in education. Additionally, our study collected data on GenAITU broadly rather than gathering information on the use of specific platforms like ChatGPT, Bing AI, or Gemini. We also conducted mediation analysis using time-lagged data, which does not establish causality, which is a key assumption in mediation. Future studies should explore the effects of technostress and technological self-efficacy in relation to GenAITU. While this study employed a time-lag design, future research could adopt longitudinal using LGM or experimental designs to more rigorously examine causal pathways and long-term effects of AI adoption on competencies and learning outcomes, yielding further valuable insights. The early stage of GenAI integration in education may limit students’ ability to evaluate its performance benefits. Digital literacy, institutional support, and exposure to AI tools likely contribute to the non-significant effects of EE, FC, and PE. Future studies should examine these factors as potential moderators to clarify their influence.

## Data Availability

The datasets generated and/or analyzed during the current study are available from the corresponding author upon reasonable request.
